# Estrogen attenuates TGF-β1-induced EMT in intrauterine adhesion by activating Wnt/β-catenin signaling pathway

**DOI:** 10.1590/1414-431X20209794

**Published:** 2020-07-06

**Authors:** Jia Cao, Dan Liu, Shiyun Zhao, Liwei Yuan, Yani Huang, Jingwen Ma, Zhijuan Yang, Bin Shi, Libin Wang, Jun Wei

**Affiliations:** 1 College of Clinical Medicine, Ningxia Medical University, Yinchuan, Ningxia, China; 2 Department of Gynecology, General Hospital of Ningxia Medical University, Yinchuan, Ningxia, China; 3 Key Laboratory of Ministry of Education for Fertility Preservation and Maintenance, Ningxia Medical University, Yinchuan, Ningxia, China; 4 Department of Beijing National Biochip Research Center Sub-Center in Ningxia, General Hospital of Ningxia Medical University, Yinchuan, Ningxia, China

**Keywords:** Intrauterine adhesion, Estrogen, Epithelial-mesenchymal transition, Wnt/β-catenin pathway

## Abstract

Although estrogen has crucial functions for endometrium growth, the specific dose and underlying molecular mechanism in intrauterine adhesion (IUA) remain unclear. In this study, we aimed to investigate the effects of estrogen on epithelial-mesenchymal transition (EMT) in normal and fibrotic endometrium, and the role of estrogen and Wnt/β-catenin signaling in the formation of endometrial fibrosis. CCK-8 and immunofluorescence assay were performed to access the proliferation of different concentrations of estrogen on normal human endometrial epithelial cells (hEECs). qRT-PCR and western blot assay were utilized to explore the effect of estrogen on EMT in normal and fibrotic endometrium, and main components of Wnt/β-catenin signaling pathway *in vitro*. Hematoxylin and eosin and Masson staining were used to evaluate the effect of estrogen on endometrial morphology and fibrosis *in vivo*. Our results indicated that the proliferation of normal hEECs was inhibited by estrogen at a concentration of 30 nM accompanied by upregulation of mesenchymal markers and downregulation of epithelial markers. Interestingly, in the model of transforming growth factor β1 (TGF-β1)-induced endometrial fibrosis, the same concentration of estrogen inhibited the process of EMT, which might be partially mediated by regulation of the Wnt/β-catenin pathway. In addition, relatively high doses of estrogen efficiently increased the number of endometrial glands and reduced the area of fibrosis as determined by the reduction of EMT in IUA animal models. Taken together, our results demonstrated that an appropriate concentration of estrogen may prevent the occurrence and development of IUA by inhibiting the TGF-β1-induced EMT and activating the Wnt/β-catenin pathway.

## Introduction

The endometrium is the inner layer of the uterus composed of epithelium and stromal components that under the effect of hormones receive embryo implantation (1,2). Intrauterine adhesion (IUA) is characterized by endometrial fibrosis. It is one of the most serious complications in patients with injuries of the endometrial basal layer, and repetitive injuries result in the formation of scar tissues that can partially or completely obstruct the uterine cavity (3). The incidence of IUA among women of reproductive age in China has increased in recent years due to trauma and infections (4). The clinical symptoms of IUA are menstrual disorders, habitual abortion, and secondary infertility (5). The current therapeutic lines mainly use hysteroscopic adhesiolysis combined with postoperative hormone therapies to prevent endometrial fibrosis and facilitate endometrial regeneration. Nevertheless, patients with severe adhesions still have a high recurrence rate and poor prognosis (6).

Although estrogen therapy has been commonly used as an important adjuvant method to prevent postoperative re-adhesions by increasing the sensitivity of the residual endometrium in the uterine cavity to estrogen receptors, there is still much controversy about the clinical application of estrogen for treating IUA. Liu et al. (7) reported that there was no significant difference in the rate of adhesions between the patients treated with estrogen at a dose of 4 or 10 mg daily; however, Liu et al. (8) found that preoperative application of a high dose of estrogen (9 mg/day) could be used as an alternative effective method for the prevention of IUA. Therefore, currently there is lack of unified standards on the dosage and administration time of estrogen, and the underlying mechanism of estrogen treatment for IUA also needs to be further elucidated.

It is well known that epithelial-mesenchymal transition (EMT), one of the most important mechanisms of fibrotic diseases, has recently been considered to be intimately involved in the pathogenesis of endometrial fibrosis (9,10). Transforming growth factor β1 (TGF-β1), an archetypical pro-inflammation and fibrosis cytokine, is related to biological processes including inflammatory activity, cell adhesion, and EMT progress (11). Previous studies reported that the mesenchymal marker vimentin is increased while epithelial marker E-cadherin is decreased in injured endometrium of IUA animal model (12). Guo et al. (13) provided specific evidence suggesting that TGF-β1/BMP7/Smad signaling is coincident with EMT in a rat IUA model. Yao et al. (14) also reported that bone marrow stem cell (BMSC)-derived exosomes are able to promote endometrium recovery by reversing EMT via a mechanism of targeting the TGF-β1/Smad pathway. These results suggest that EMT is likely to be one of the main mechanisms of endometrial repair disorder in IUA. Inhibiting EMT may be therefore a novel strategy for treatment of IUA.

The Wnt signal is composed of highly conserved and secreted glycoproteins, which play key roles in many biological processes. At present, several lines of evidence have demonstrated that an aberrant expression of important regulatory proteins in Wnt/β-catenin signaling is closely related to the occurrence of fibrotic diseases (15). In this regard, Akhmetshina et al. (16) reported that silencing β-catenin could attenuate TGF-β-induced fibrosis in endometriosis. In addition, Van Der Horst et al. (17) proved that estrogen has been introduced to target Wnt/β-catenin pathway to regulate the growth of endometrial epithelial cells, and the interaction between estrogen and Wnt/β-catenin signaling is one of the important mechanisms to maintain endometrial homeostasis. These findings clearly demonstrated that the relationship between estrogen and Wnt/β-catenin signaling plays an important role in the development of IUA. However, the impact and mechanism of estrogen on EMT and alteration of Wnt/β-catenin signaling in endometrial fibrosis remain unclear.

Therefore, in this study, we first attempted to investigate the effect of estrogen on the outcome of EMT in normal endometrial glandular epithelial cells and fibrotic cells, and then explored the correlation between estrogen on TGF-β1-induced EMT and abnormal activation of Wnt/β-catenin signaling in an IUA cell model. Our findings may provide an insight into the long-term estrogen application and clinical treatment for IUA.

## Material and Methods

### Human tissue collection

Biopsies of human endometrium samples were obtained from the women undergoing hysteroscopy in the General Hospital of Ningxia Medical University. Tissues from thirty donors were collected and analyzed in this study. The endometrium was scraped off and collected into D-Hanks phosphate buffer solution (PBS), and was immediately used for cell isolation. The sample was collected after informed patient consent and the study was approved by the Ethics Committee of Scientific Research of the General Hospital of Ningxia Medical University (2018-058).

### Isolation and culture of human endometrial epithelial cells (hEECs)

Briefly, the endometrial tissues of healthy adult women were collected and immersed in PBS solution containing 100 U/mL penicillin and 100 mg/mL streptomycin. The tissue was minced with sterile scissors into small pieces for isolation of hEECs. After removing red blood cells, the pieces were collected into a centrifuge tube and washed with cold PBS solution, and directly digested with dissociation buffer containing HBSS buffer (Gibco, USA) and 3.0 mg/mL collagenase type IV (Gibco) for 10 min at 37°C with gentle agitation. Then, the same volume of Accumax (Innovative Cell Technologies, USA) was added in the dissociated solution and incubated at 37°C for an additional 10 min for further digestion. Dissociated cells were filtered through a 400-mesh nylon sieve to remove cell debris. The cell suspension was centrifuged at 1000 *g* for 10 min at room temperature and the cell pellet was washed with cold PBS prior to being pelleted for collection of glandular epithelial cells. The cells were resuspended in PneumaCult™-Ex Plus Complete Medium (Stem Cell, Canada) and seeded onto petri dish pre-coated with collagen type I from rat tail (Millipore, USA) at 37°C and 5% CO_2_. The hEEC colonies emerged after 2 to 3 days and were dissociated using Accutase solution (Sigma, USA) for cell culture expansion.

### RNA extraction and quantitative real-time PCR

Total RNA was extracted from hEEC using TRIzol reagent (Invitrogen, USA). The concentration of extracted RNA was measured by Nanodrop (ThermoFisher Scientific, USA). cDNA synthesis was performed from 1 μg RNA using a reverse transcription kit (TaKaRa, China), according to the manufacturer's protocols. Real-time PCR amplification was performed with specific primers and carried out using the SYBR-Green PCR system (Takara Bio, Inc.). PCR amplification was carried out as follows: 95°C for 30 s, followed by 40 cycles of 95°C for 5 s and 60°C for 30 s. β-actin served as reference gene for mRNA normalization. The relative expression of each gene was quantified by the 2^−ΔΔCt^ method. The primers used in this study are listed in Table 1.

**Table 1:** Primer sequences used for qRT-PCR analysis.

Genes	Access number	Location	Forward primer	Reverse primer
β-actin	NM_001101.5	767−897	CCACGGCTGCTTCCAGCTCC	GGACTCCATGCCCAGGAAGGAA
ERα	NM_000125.4	1262−1390	GCTTACTGACCAACCTGGCAGA	GGATCTCTAGCCAGGCACATTC
CK8	NM_001256282.2	714−803	TACATGAACAAGGTAGAGCTGG	CCGGATCTCCTCTTCATATAGC
CK18	NM_000224.3	691−873	TCATGAAGAAGAACCACGAAGA	GAGACCAGTACTTGTCTAGCTC
EPCAM	NM_002354.3	277−364	GTCTGTGAAAACTACAAGCTGG	CAGTATTTTGTGCACCAACTGA
E-cadherin	NM_001317185.2	1216−1423	AGTCACTGACACCAACGATAAT	ATCGTTGTTCACTGGATTTGTG
FOXA2	NM_021784.5	1410−1543	GGAACACCACTACGCCTTCAAC	AGTGCATCACCTGTTCGTAGGC
MUC1	NM_001018016.3	689−833	CCTACCATCCTATGAGCGAGTAC	GCTGGGTTTGTGTAAGAGAGGC
N-cadherin	NM_001308176.2	2196−2356	CATCATCCTGCTTATCCTTGTG	CATAGTCCTGGTCTTCTTCTCC
Vimentin	NM_003380.5	100−193	AAACTTAGGGGCGCTCTTGT	CGCTGCTAGTTCTCAGTGCT
ZEB1	NM_001128128.3	895−994	TTACACCTTTGCATACAGAACCC	TTTACGATTACACCCAGACTGC

The species used was human.

### Cell viability analysis

Cell viability was detected using the Cell Counting Kit-8 (CCK8) (KeyGEN BioTECH, China), according to the manufacturer's instructions. Briefly, a total of 2×10^3^ endometrial epithelial cells were plated into 96-well plates and allowed to attach overnight at 37°C. Estrogen stock solution was added to the plates and the cells were cultured in the presence of various final concentrations (10, 30, 50 nM) for indicated times (24, 48, 72 h). Then, 10 µL CCK8 solution was added to each well and incubated at 37°C for 2 h, and absorbance was readout at 450 nm using a microplate reader (Glomax Multi Detection System, Promega, USA) to determine the cell viability.

### Western blotting

Cells were harvested and lysed using RIPA lysis buffer (Beyotime Biotechnology, China) supplemented with protease inhibitor cocktail (Roche, USA) for 45 min on ice. Then, the lysates were centrifuged at 13,000 *g* for 20 min at 4°C and protein concentration was measured by BCA protein reagent kit (ThermoFisher Scientific). Equal amounts of proteins were electrophoresed on 10% SDS-PAGE and transferred to PVDF membranes. The membranes were blocked with 5% defatted milk for 1 h at room temperature and incubated with specific primary antibodies overnight. Subsequently, appropriate HRP-conjugated secondary antibodies were incubated at room temperature for 1 h. Finally, the proteins of interest were visualized using ECL in the BioImaging System (BIO-RAD, USA). GAPDH was used as an internal control to normalize the relative expression of each protein of interest. The primary antibodies used in this study are listed in Table 2.

**Table 2:** Antibodies used in this study.

Antibody	Catalog Number	Company	Specificity	Antibody dilution	
				WB	IF
GAPDH	ab128915	Abcam	Rabbit monoclonal	1:5000	1:200
CK8	ab9023	Abcam	Mouse monoclonal	1:1000	1:200
CK7	ab181598	Abcam	Rabbit monoclonal	1:1000	1:200
EPCAM	ab71916	Abcam	Rabbit polyclonal	1:1000	1:200
E-cadherin	#3195	Cell Signaling Technology	Rabbit monoclonal	1:1000	1:500
Vimentin	ab92547	Abcam	Rabbit monoclonal	1:1000	
ERα	ab32063	Abcam	Rabbit monoclonal	1:1000	
N-cadherin	ab76011	Abcam	Rabbit monoclonal	1:2000	
Smad3	ab40854	Abcam	Rabbit monoclonal	1:1000	
p-Smad3	ab52903	Abcam	Rabbit monoclonal	1:2000	
Smad2	ab40855	Abcam	Rabbit monoclonal	1:2000	
p-Smad2	#18338T	Cell Signaling Technology	Rabbit monoclonal	1:1000	
p-keratin	ab8068	Abcam	Mouse monoclonal	1:1000	
Collagen I	ab34710	Abcam	Rabbit polyclonal	1:1000	
CyclinD1	ab134175	Abcam	Rabbit monoclonal	1:2000	
C-myc	ab32072	Abcam	Rabbit monoclonal	1:1000	
MMP9	ab76003	Abcam	Rabbit monoclonal	1:2000	
β-catenin	NBP1-32239	NOVUS	Rabbit polyclonal	1:2000	
GSK3β	ab32391	Abcam	Rabbit monoclonal	1:5000	
Axin2	ab109307	Abcam	Rabbit monoclonal	1:2000	
c-jun	#9165	Cell Signaling Technology	Rabbit monoclonal	1:1000	

WB: Western blotting; IF: immunofluorescence.

### Immunofluorescence staining

For the immunofluorescence (IF) staining, cells cultured on cover slides were fixed in 4% paraformaldehyde at room temperature for 20 min, and then incubated for 10 min with 0.3% Triton X-100 to improve cell permeability. Subsequently, the cells were blocked with 5% normal goat serum (ThermoFisher Scientific) at room temperature to block the non-specific binding. Primary antibodies used in this work included epithelial cell markers CK7, CK8, EPCAM, and E-cadherin, and human specific marker Lamin A/C (1:200, #4777, Cell Signaling Technology, USA). Detailed information of antibodies is shown in Table 2. Alexa Fluor-conjugated Donkey 488/594 (1:200, Life Technologies, USA) were used as secondary antibodies. The nucleus was visualized with DAPI staining (Sigma) for 15 min in the dark and then mounted with fluorescence quenching agent (Solarbio, USA). Images were taken under a fluorescence microscope (Olympus, Japan).

### Generation of IUA rabbit model

New Zealand female rabbits weighing about 2.0-2.5 kg were purchased from Xi'an Bioscience Co. Ltd. (China), and all rabbits were given a basic diet for one week to adapt to the laboratory environment. Twenty-five rabbits were randomly divided into five groups, including Control group, Sham group, IUA model group, E2 (0.1 mg/kg estrogen), and E2 (0.5 mg/kg estrogen). The IUA animal model was generated by a dual damage method of mechanical curettage and lipopolysaccharide (LPS, 6 mg/L, Sigma) infection. The rabbits in the Control and Sham groups received no treatment, those in the estrogen groups received intramuscular injections of estrogen for 20 days, while the IUA model group received PBS as a control.

### H&E staining and Masson staining

The endometrial tissue samples were fixed with 4% paraformaldehyde for 24 h and then embedded into paraffin blocks after dehydration and hyalinization. The paraffin-embedded tissues were cut into 5-μm-thick slices for hematoxylin and eosin (H&E) histochemical staining to evaluate the alterations of endometrial morphology. Modified Masson's trichrome staining was performed using a kit (Solarbio) to assess the extent of endometrial fibrosis. Five fields of vision were randomly selected for each slide under the microscope (Olympus), and the statistical differences were analyzed using Image Pro Plus 6.0 software (Media Cybernetics, USA).

### Immunohistochemical staining

Samples were fixed in 4% paraformaldehyde and embedded in paraffin. The transverse paraffin sections were deparaffinized using xylene and rehydrated through a decreased gradient concentrations of alcohol solution. Then, the sections were incubated in 3% hydrogen peroxide for 30 min to inactivate endogenous peroxidase and incubated with the following primary antibodies: anti-ERα (Cell Signaling Technology, 1:200), anti-E-cadherin (Cell Signaling Technology, 1:400), anti-vimentin (Abcam, USA, 1:400) at 37°C for 2 h. Subsequently, the biotinylated secondary antibody was incubated for 1 h at 37°C, followed by addition of 3,3'-diaminobenzidine to visualize the reaction products. The number of positively stained cells and absorbance were quantified at five randomly selected fields per section.

### Statistical analysis

Statistical analysis was performed using SPSS 20.0 (IBM, USA) and GraphPad Prism version 6.0 (USA). The data are reported as means±SD. Two samples were compared using independent sample two-tailed *t*-test. Statistical differences among multiple groups were determined by one-way analysis of variance (ANOVA). P<0.05 was considered to be statistically significant.

## Results

### Cultivation and characterization of primary hEECs

**Figure 1:**
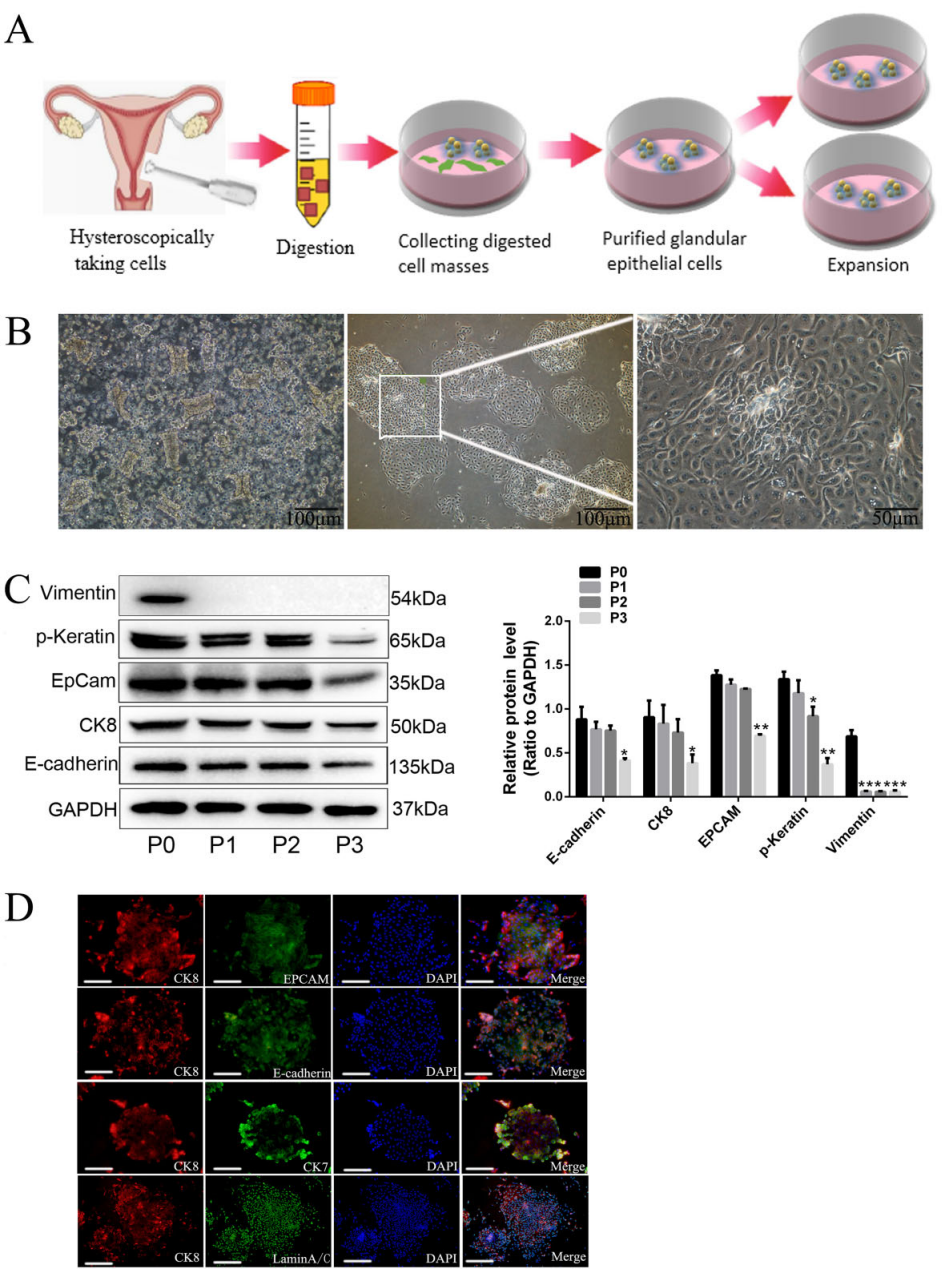
Cultivation and characterization of human endometrial epithelial cells (hEECs). **A**, Diagram showing the procedure of hEECs isolation and expansion. **B**, The left panel shows hEECs without adherence (scale bar 100 μm); the middle and right panels show images of attached hEECs at different magnifications after 24 h (scale bars 100 and 50 μm). **C**, Western blotting showing the protein levels of endometrial epithelial markers in P0 to P3 generations. **D**, Anti-CK8, anti-epithelial cell adhesion molecule (EPCAM), anti-E-cadherin, anti-CK7, and anti-laminA/C immunostaining of hEECs colonies with nuclei counterstain (magnification 200×, scale bar 50 μm). Data are reported as means±SD. *P<0.05, **P<0.01 compared to P0 (ANOVA).

Primary hEECs were isolated from normal female endometrium and purified according to a previously described method with slight modifications (18). The workflow of hEECs isolation and expansion is summarized in Figure 1A. Microphotographs of hEECs cultured onto collagen type I rat tail-coated dishes showed a marble morphology of epithelial cells (Figure 1B). The primary culture of cells expressed epithelial cell markers, such as cytokeratin 8 (CK8), epithelial cell adhesion molecule (EPCAM), E-cadherin and p-keratin, but not vimentin as determined by immunoblotting assay (Figure 1C). The colonies with morphology of glandular epithelial cells were further corroborated by immunofluorescence staining for the antibodies against the epithelial cell markers: CK7, CK8, EPCAM, and E-cadherin, and human-derived marker laminA/C (Figure 1D). These results indicated that human endometrial glandular epithelial cells had been successfully isolated and expanded in culture.

### Estrogen regulated the epithelial-mesenchymal transition (EMT) in normal hEECs

**Figure 2:**
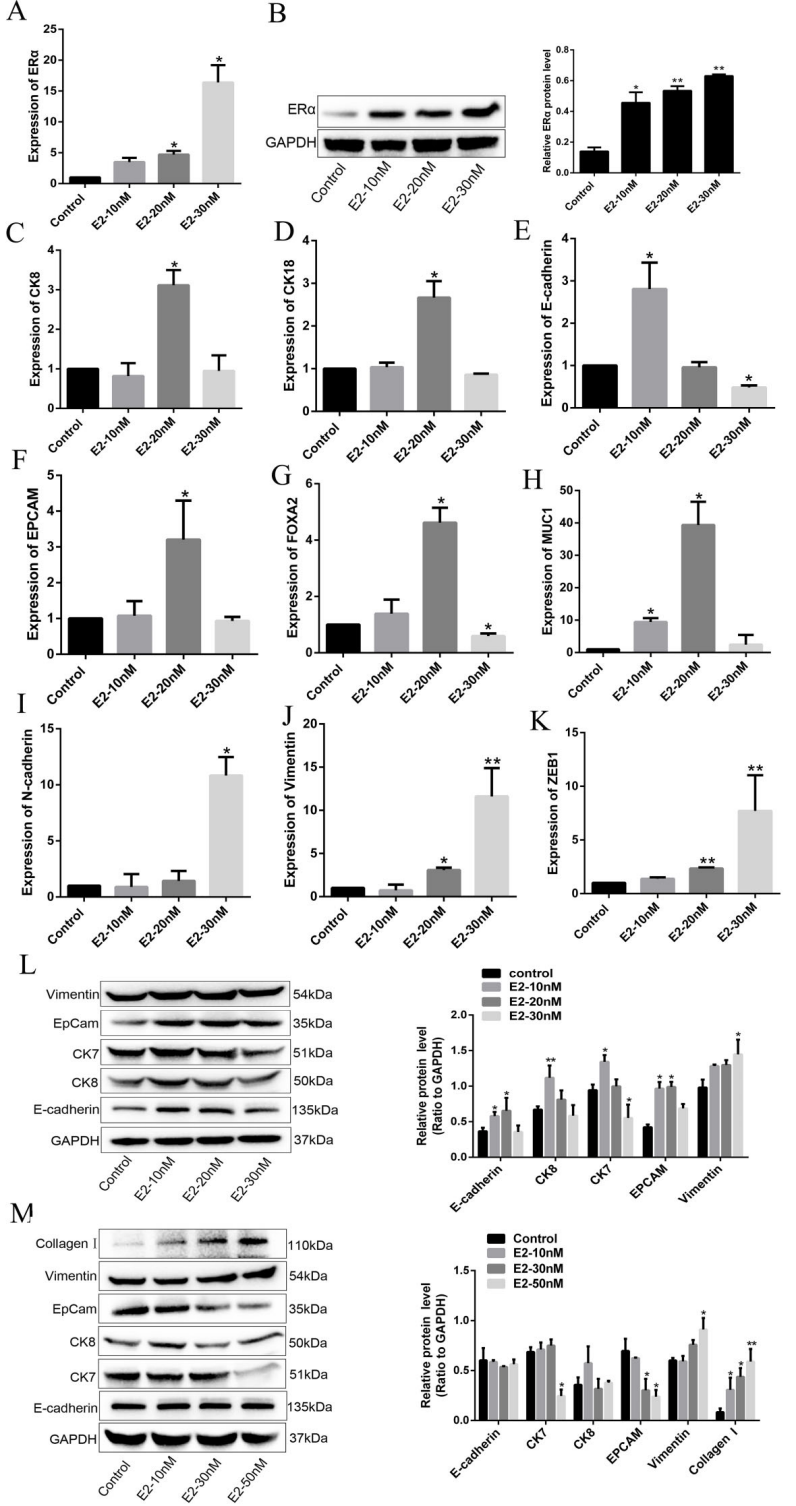
Effects of different doses of estrogen (E2) (10, 20, 30 nM) on epithelial-mesenchymal transition (EMT)-related markers in normal human endometrial epithelial cells (hEECs). **A**, RT-PCR showing the mRNA expression of estrogen receptor α (ERα). **B**, Western blotting showing the protein expression of ERα. **C**–**E**, mRNA expression levels of CK8, CK18, and E-cadherin. **F**–**H**, mRNA expression levels of anti-epithelial cell adhesion molecule (EPCAM), FOXA2, and MUC1. **I**–**K**, mRNA expression levels of mesenchymal markers N-cadherin, vimentin, and ZEB1. **L** and **M**, Protein expression of assessed EMT-related markers detected by western blotting with different doses of E2 (10, 20, 30 nM and 10, 30, 50 nM, respectively). Data are reported as means±SD. *P<0.05, **P<0.01 compared to control (ANOVA).

To determine the impact of estrogen on EMT progression, the expression of epithelial and mesenchymal markers was detected in normal hEECs treated with different concentrations of estrogen (10, 20, 30 nM). Firstly, we detected the expression level of estrogen receptor α (ERα), and found that ERα expression was increased in a concentration-dependent manner as assessed by the levels of transcript and protein, compared to the control group (Figure 2A and B). In contrast, the expression of transcripts of epithelial markers, including CK8, CK18, E-cadherin, EPCAM, FOXA2, and MUC1, were decreased significantly in cells treated with 30 nM of estrogen compared to lower concentrations of estrogen (10 and 20 nM) and the control group (Figure 2C–H). However, the expression of mesenchymal markers, such as vimentin, N-cadherin, and ZEB1, was significantly increased in cells exposed to 30 nM of estrogen (Figure 2I–K). These results were further confirmed by western blotting analysis. The expression of epithelial markers was decreased and the expression of mesenchymal markers was increased in normal hEECs treated with 30 nM of estrogen, which was consistent with the RT-PCR results (Figure 2L). Interestingly, similar results were observed in cells treated with estrogen at a concentration of 50 nM (Figure 2M). Taken together, these results suggested that relatively high doses of estrogen (30 nM) could promote EMT in normal hEECs.

### Cell proliferation of normal hEECs at different concentrations of estrogen

**Figure 3:**
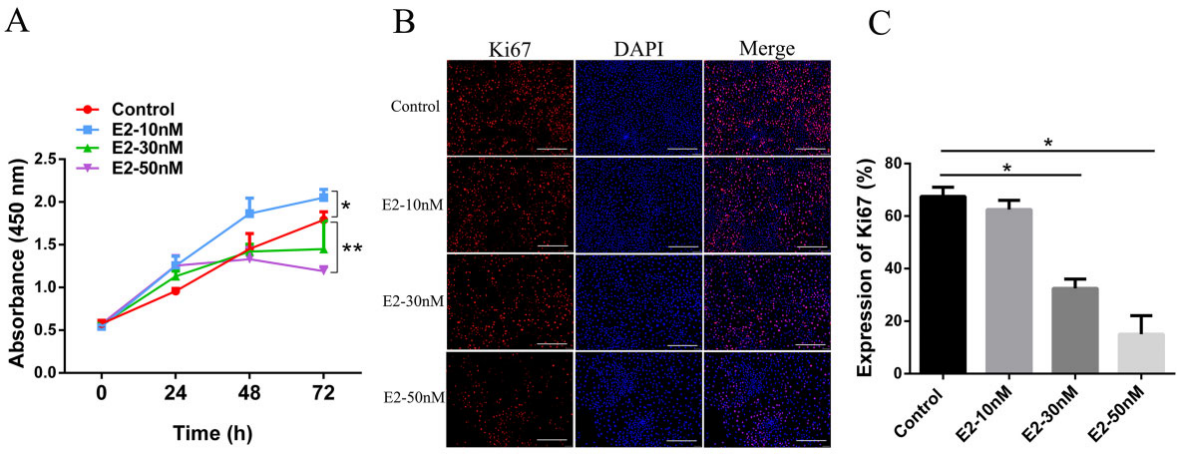
**A**, Cell proliferation at different times was detected by CCK8 assay in normal human endometrial epithelial cells. **B** and **C**, Detection of Ki67 expression by immunofluorescence staining of human endometrial epithelial cells treated with different doses of estrogen (E2) (magnification 100×, scale bar 100 μm). Data are reported as means±SD. *P<0.05, **P<0.01 (ANOVA).

Next, we examined the effects of estrogen on normal hEECs proliferation at different concentrations (10, 30 and 50 nM). Cell viability was significantly increased in culture with 10 nM of estrogen, whereas it was decreased in cells exposed to a higher concentration of estrogen (30 and 50 nM), especially at 72 h post-estrogen exposure (Figure 3A). The above results were further confirmed by assessing the expression of proliferative marker Ki67 as determined by IF staining. The proliferation rate was decreased significantly in the cells treated with 30 or 50 nM of estrogen (Figure 3B and C). Taken together, these findings suggested a dose-dependent effect of estrogen on hEECs, i.e., a greater than 30 nM of estrogen could inhibit the proliferation of normal hEECs by promoting EMT progression.

### Estrogen inhibited the TGF-β1-induced EMT in IUA

**Figure 4:**
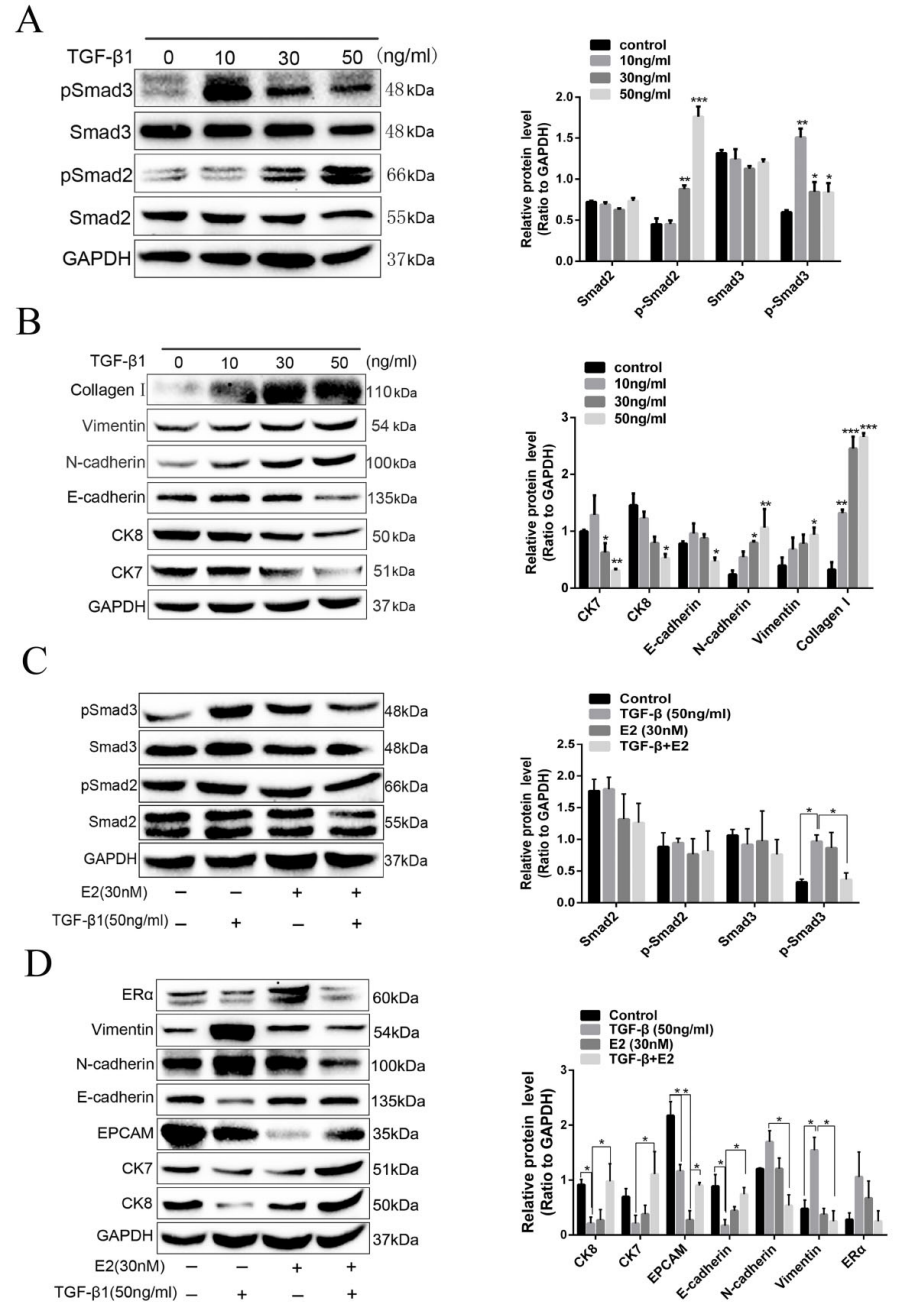
Influence of estrogen on transforming growth factor β1 (TGF-β1)-induced epithelial-mesenchymal transition (EMT) in intrauterine adhesion. **A**, Human endometrial epithelial cells were treated with TGF-β1 for 24 h to detect the total and phosphorylation protein levels of Smad2 and Smad3 by western blotting. **B**, Expression of fibrotic and EMT markers was determined in cells at 24 h post-stimulation of TGF-β1. **C**, The total and phosphorylation protein levels of Smad2 and Smad3 were detected in cells incubated with estrogen (E2, 30 nM) for 48 h. **D**, The expression of EMT markers in the TGF-β-induced cells treated with E2 was detected by western blotting. Data are reported as means±SD. *P<0.05, **P<0.01, ***P<0.001 (ANOVA).

It has been reported that TGF-β is a well-established central mediator of endometrial fibrosis, and TGF-β/Smad signaling pathway plays critical roles in the pathogenesis of IUA (19). In the present study, a cell model of IUA was generated by exposing hEECs to TGF-β1. Indeed, induction by different concentrations of TGF-β1 (10, 30, 50 ng/mL) for 24 h led to activation of TGF-β/Smad signaling in hEECs, mainly manifested as a significantly increased expression of phosphorylation Smad3 and Smad2 (Figure 4A). In addition, the TGF-β1 induction also increased the expression of collagen I, N-cadherin, and vimentin, whereas it decreased the expression of E-cadherin, CK7, and CK8 in a concentration-dependent manner (Figure 4B). The above results suggested that TGF-β1 induced EMT and endometrial fibrosis progression by activating the TGF-β/Smad signaling pathway in IUA. By using the TGF-β1-induced fibrosis cell model, the biological function of estrogen on TGF-β/Smad signaling and EMT progression in IUA were further investigated. As expected, a decrease of TGF-β1-induced Smad3 phosphorylation was observed in hEECs pretreated with 30 nM of estrogen for 48 h, but Smad2 phosphorylation expression was not altered (Figure 4C). Furthermore, a reduced expression of mesenchymal markers but an increased expression of epithelial markers were observed in TGF-β1-induced fibrosis cells that were pretreated with 30 nM of estrogen (Figure 4D). These results indicated that estrogen might inhibit EMT progression in endometrial fibrosis and prevent the initiation of IUA by targeting the TGF-β/Smad3 signaling.

### Involvement of Wnt/&mac_bgr;-catenin signaling in estrogen-inhibited EMT progression in IUA

**Figure 5:**
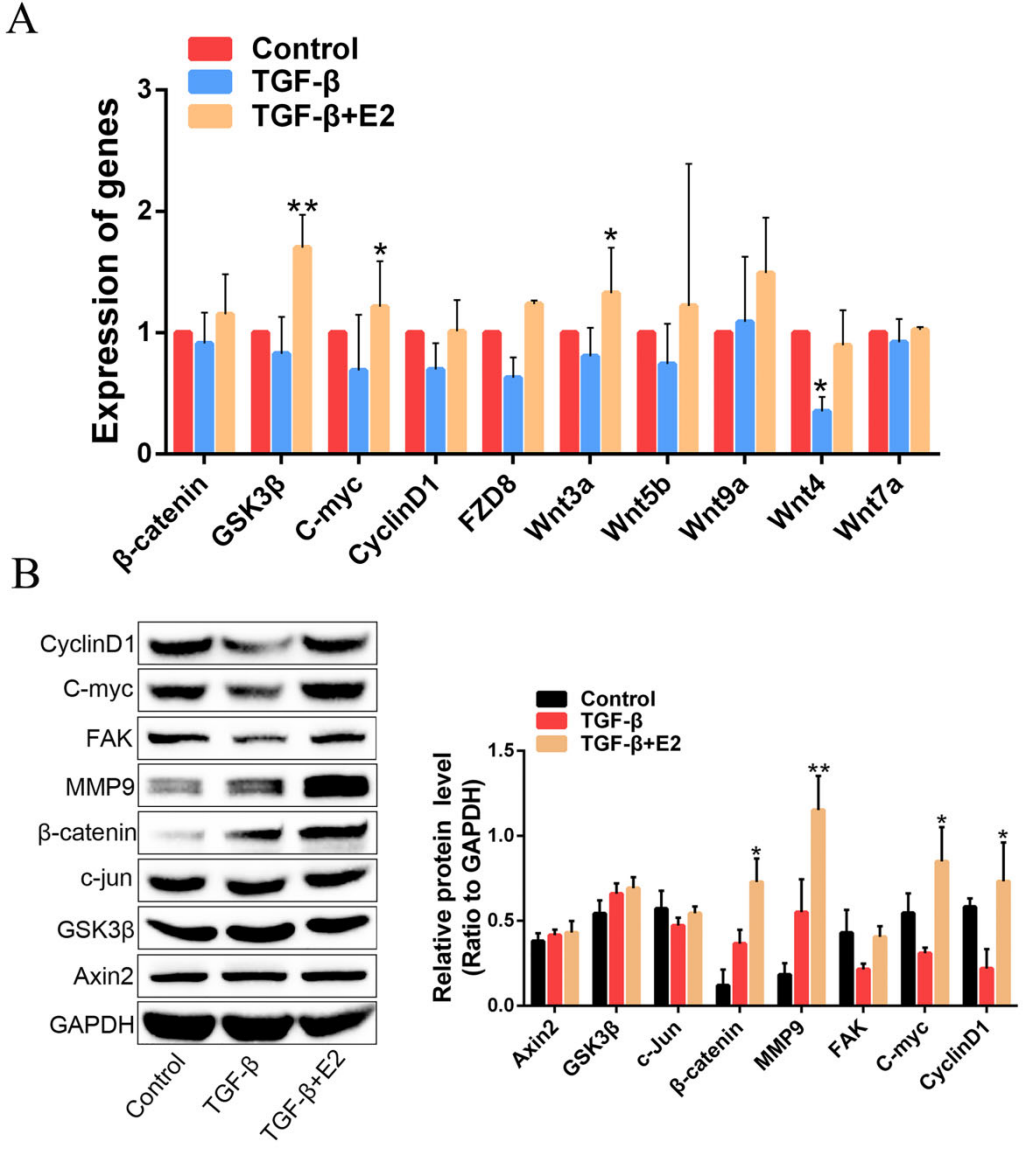
Estrogen (E2, 30 nM) inhibited intrauterine adhesion progression through activating the Wnt/β-catenin signaling pathway. **A**, Expression levels of key components of Wnt/β-catenin pathway were detected by RT-PCR assay. **B**, Protein expression levels of Wnt/β-catenin pathway were analyzed by western blotting. Data are reported as means±SD. *P<0.05, **P<0.01 compared to control (ANOVA).

The function of Wnt/β-catenin signaling in IUA pathogenesis has been established. In order to determine whether Wnt/β-catenin signaling was involved in the estrogen-inhibited EMT in the IUA progression, the key components of Wnt/β-catenin signaling were assessed by RT-PCR analysis. mRNA expression levels of key molecules, including β-catenin, GSK3β, C-myc, cyclinD1, FZD8, and other ligands (Wnt3a, Wnt5b, Wnt9a, Wnt4, Wnt7a), were decreased in TGF-β1-induced hEECs, whereas the above molecules expressions were increased when estrogen was added to TGF-β1-induced fibrosis cells (Figure 5A). Furthermore, we confirmed the results by immunoblotting assay, and found that the abundance of proteins β-catenin, MMP9, FAK, C-myc, and cyclinD1 in the estrogen-treated group were significantly increased compared to the TGF-β1-induced group and the control group, which was in agreement with the mRNA expression (Figure 5B). Collectively, these results demonstrated that estrogen inhibited TGF-β1-induced EMT in IUA progression, which was in part through activating the Wnt/β-catenin signaling.

### Relatively high doses of estrogen restored endometrial morphology in a rabbit model of IUA

**Figure 6:**
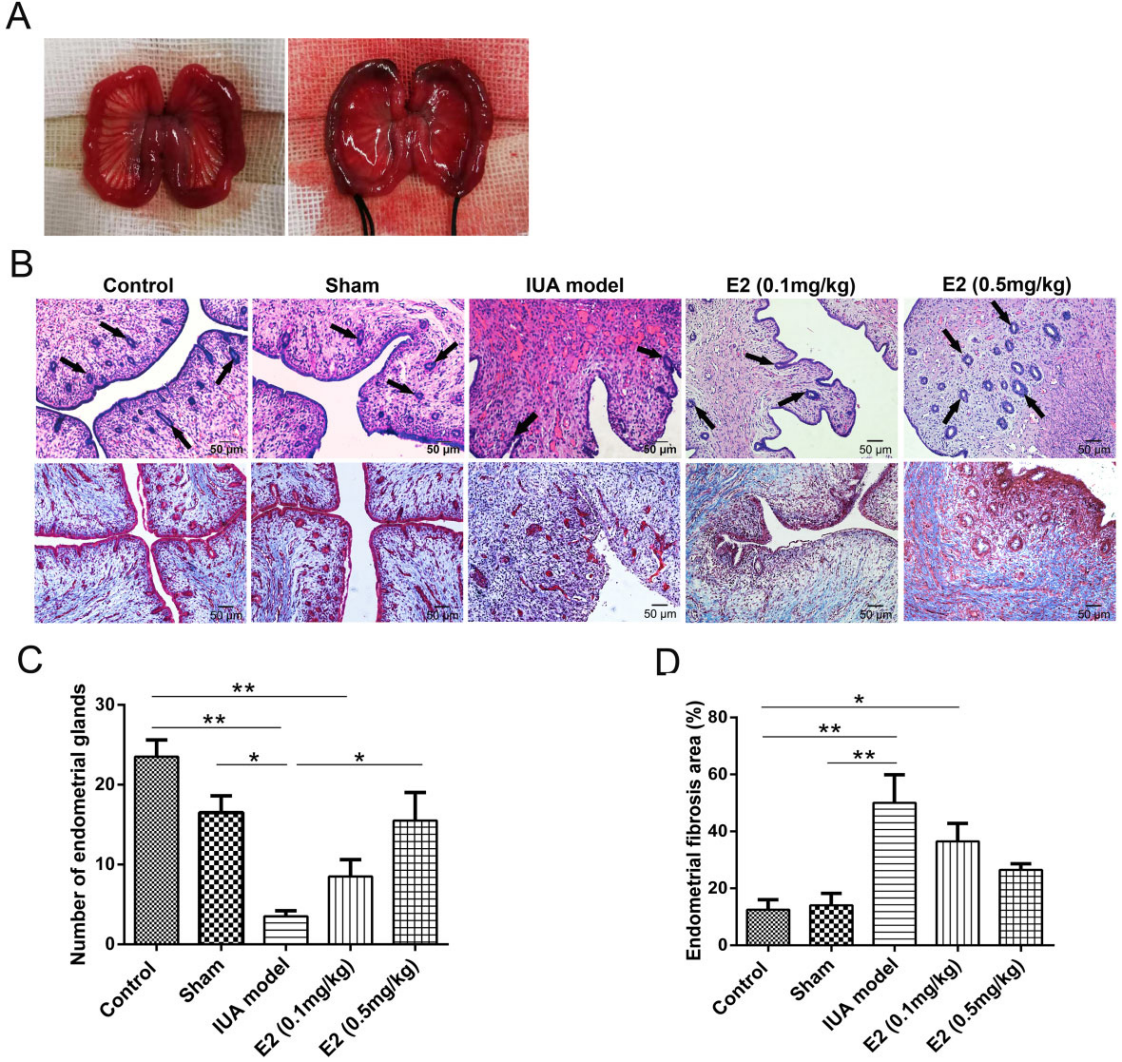
Relatively high doses of estrogen (E2) for the treatment of a rabbit intrauterine adhesion (IUA) model. **A**, Normal morphology of rabbit uterus (left) and of rabbit uterus damaged by mechanical injury and lipopolysaccharide infection (right). **B**, Endometrial morphology was observed by hematoxylin and eosin and Masson staining (magnification 200×, scale bar 50 μm). **C**, Statistical comparison of endometrial glands in the visual field of each group. **D**, Statistical results of endometrial fibrosis area in each group. Data are reported as means±SD. *P<0.05, **P<0.01 (ANOVA).

**Figure 7:**
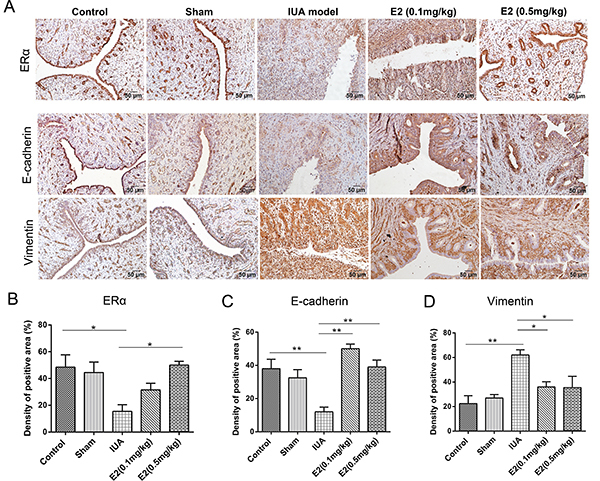
Impact of estrogen (E2) on epithelial-mesenchymal transition in a rabbit intrauterine adhesion (IUA) model. **A**, Representative images for localization of ERα, E-cadherin, and vimentin in the endometrium tissues of each group by immunohistochemical staining (magnification 200×, scale bar 50 μm). The expression levels of ERα (**B**), E-cadherin (**C**), and vimentin (**D**) were semi-quantified by area of positive staining. Data are reported as means±SD. *P<0.05, **P<0.01 (ANOVA).

In order to verify the dose-dependent effect of estrogen on IUA treatment *in vivo*, a New Zealand rabbit model of IUA was constructed using mechanical and infection double injury methods (Figure 6A). The rabbits in treatment groups were intramuscularly injected with 0.1 and 0.5 mg/kg estrogen, and the IUA group was given to the same volume of PBS. According to H&E staining results (Figure 6B and C), the uterine cavity presented bleeding and inflammatory infiltration, as well as a decreased number of endometrial glands in the IUA group, compared with the control and sham operation groups. However, the endometrial morphology significantly improved and glandular numbers increased as the estrogen concentration increased. Masson staining was used to assess the extent of endometrial fibrosis. Compared with the control and sham groups, the area of endometrial fibrosis increased in the IUA model group, while the fibrotic area gradually decreased after estrogen treatment (Figure 6B and D). To further detect the effect of estrogen on EMT *in vivo*, the expression of ERα, E-cadherin, and vimentin was evaluated by immunohistochemical staining. The expression of ERα and E-cadherin was decreased and expression of vimentin was increased in the IUA model group. However, an increased expression of ERα and E-cadherin and a decreased expression of vimentin were observed in animals treated with estrogen (Figure 7A–D). Taken together, these results showed that relatively high doses of estrogen were more effective for IUA treatment through inhibiting the EMT process.

## Discussion

IUA is a medical condition defined by the abnormal presence of endometrial tissues within the adhesions and the main mechanisms include endometrial basal layer damage, endometrial repair disorders, and fibrosis healing (20). Although several treatment options including hysteroscopic adhesiolysis combined with intrauterine device and estrogen and progesterone have been effective for IUA, its incidence and recurrence rates are still notably high (21). Therefore, it is important to delineate the molecular mechanisms underlying the progression of this disease.

In this study, we elucidated the influence of estrogen on the establishment and maintenance of EMT in normal endometrium and fibrotic endometrium induced by TGF-β1. The induction system is a useful approach to simulate the fibrosis progression in the IUA microenvironment. Increased expression of TGF-β has been reported to be closely related to poor prognosis of multiple diseases (22). In addition, TGF-β is intimately linked to the initiation of EMT that plays a predominant role in fibrosis disease (23). However, the function of estrogen on TGF-β-induced EMT in IUA remains unclear. Our study focused on determining the effect and mechanism of estrogen on EMT in fibrotic endometrium.

First, our results demonstrated that different concentrations of estrogen had different effects on normal endometrial epithelial cells, and relatively high doses of estrogen (30 nM) inhibited cell proliferation by promoting the progress of EMT in normal endometrium. Previous studies found that estrogen was sensitive to EMT progression in endometrial cancer and endometriosis (24,25). Since endometrial fibrosis is the main cause of IUA occurrence and poor prognosis, we further investigated the impact of the same dose of estrogen on EMT in the fibrotic environment. Accumulating evidence suggests that TGF-β is the main contributor to the association between EMT and poor clinical outcome in fibrosis diseases (26,27). Similarly, in this study, the normal hEECs were treated with TGF-β1 to induce an endometrial fibrosis cell model and simulate the IUA microenvironment. The results indicated that TGF-β1 stimulation successfully induced EMT by activating TGF-β/Smad signaling. To further analyze the effect of estrogen on EMT in the context of fibrosis, hEECs were treated with TGF-β1 prior to being exposed to 30 nM of estrogen. Interestingly, the expression of CK7, CK8, EPCAM, and E-cadherin was increased, while the expression of N-cadherin, vimentin, and p-Smad3 was decreased in the estrogen-treated cells, which indicated that estrogen could reverse EMT occurrence by blocking the TGF-β/Smad3 signaling. In line with these data, our study demonstrated that a relatively high dose of estrogen might play a completely opposite role in the endometrial physiological and pathological conditions. In support of our results, previous studies have shown that different levels of estrogen have different effects on the degree of endometrial repair and stroma fibrosis in a rabbit IUA model (28). Taken together, our finding provided further insight into the potential role of estrogen to inhibit the development of EMT and promote the endometrium regeneration in IUA.

Previous studies have shown the interrelationship between estrogen and Wnt/β-catenin signaling in endometrial fibrosis (29). Zhu et al. (30) also described that Hippo and Wnt signaling pathways could form a complex signaling network with TGF-β signaling pathway to mediate endometrial fibrosis. Although the importance of estrogen in the treatment of IUA has been emphasized, the existing research has not clearly elucidated the molecular mechanism of Wnt/β-catenin signaling pathway involved in the process of estrogen-inhibited EMT in IUA. In this study, our data indicated that the protein and mRNA expression of β-catenin, GSK3β, C-myc, and cyclinD1 in the TGF-β1 induced group were downregulated compared to the control group. The above targets were significantly upregulated in the estrogen-treated group, suggesting that estrogen reversed EMT by activating the Wnt/β-catenin signaling pathway. The Wnt/β-catenin pathway is well known for its regulation of cell growth and proliferation in response to different statuses (31). Our study focused on the interaction between estrogen-reversed EMT and Wnt/β-catenin signaling in the injured endometrium, suggesting that estrogen functioned partly through regulation of this pathway.

**Figure 8:**
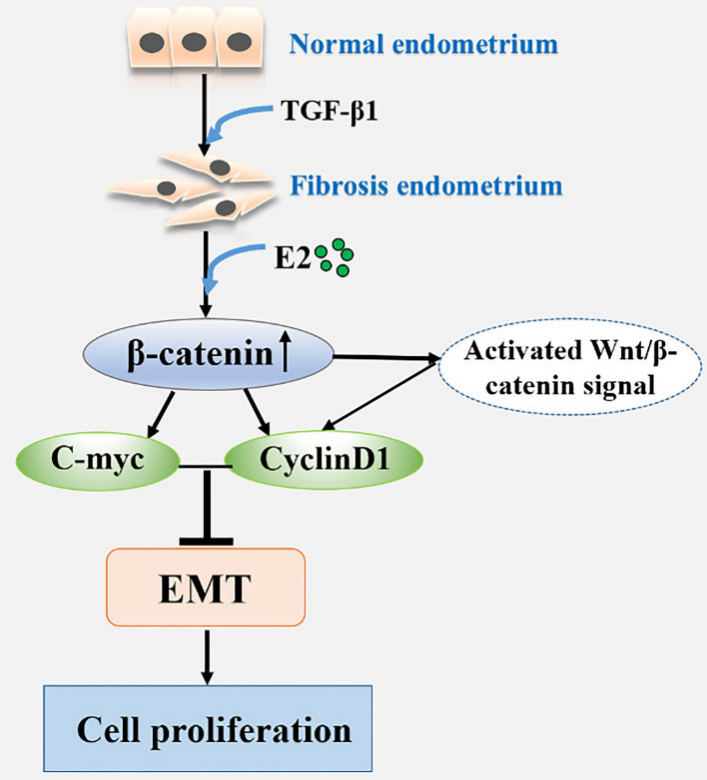
Schematic representation of Wnt/β-catenin signaling involved in the regulation of the estrogen-inhibited epithelial-mesenchymal transition (EMT) process in fibrosis endometrium.

In conclusion, our study demonstrated that the function of estrogen in endometrium regeneration could occur by inhibiting the TGF-β1-induced EMT and activating the Wnt/β-catenin signaling in the development of IUA (Figure 8). Our results also highlighted the importance of determining the appropriate concentration of estrogen for IUA treatment. These findings may provide new ideas to study the role of estrogen and illustrate the molecular mechanism of estrogen therapy.

## References

[1] Cha J, Sun X, Dey Sk (2012). Mechanisms of implantation: strategies for successful
pregnancy. Nat Med.

[2] Ruan YC, Chen H, Chan HC (2014). Ion channels in the endometrium: regulation of endometrial
receptivity and embryo implantation. Human Reprod Update.

[3] Hooker A, Fraenk D, Brölmann H, Huirne J (2016). Prevalence of intrauterine adhesions after termination of
pregnancy: a systematic review. Eur J Contracept Reprod Health Care.

[4] Dalton VK, Saunders NA, Harris LH, Williams JA, Lebovic DI (2006). Intrauterine adhesions after manual vacuum aspiration for early
pregnancy failure. Fertil Steril.

[5] Deans R, Abbott J (2010). Review of intrauterine adhesions. J Minim Invasive Gynecol.

[6] Chen L, Zhang H, Wang Q, Xie F, Gao S, Song Y (2017). Reproductive outcomes in patients with intrauterine adhesions
following hysteroscopic adhesiolysis: experience from the largest women's
hospital in China. J Minim Invasive Gynecol.

[7] Liu L, Huang X, Xia E, Zhang X, Li TC, Liu Y (2019). A cohort study comparing 4 mg and 10 mg daily doses of
postoperative oestradiol therapy to prevent adhesion reformation after
hysteroscopic adhesiolysis. Human Fertil (Camb).

[8] Liu AZ, Zhao HG, Gao Y, Liu M, Guo BZ (2016). Effectiveness of estrogen treatment before transcervical
resection of adhesions on moderate and severe uterine adhesion
patients. Gynecol Endocrinol.

[9] Lin X, Chai G, Wu Y, Li J, Chen F, Liu J (2019). RNA M^6^A methylation regulates the epithelial
mesenchymal transition of cancer cells and translation of
Snail. Nat Commun.

[10] Wang P, Luo Ml, Song E, Zhou Z, Ma T, Wang J (2018). Long noncoding RNA inhibits renal fibrogenesis by negatively
regulating the TGF-β/Smad3 pathway. Sci Transl Med.

[11] Choi HJ, Park MJ, Kim BS, Choi HJ, Joo B, Lee KS (2017). Transforming growth factor β1 enhances adhesion of endometrial
cells to mesothelium by regulating integrin expression. BMB Rep.

[12] Xu Q, Duan H, Gan L, Liu X, Chen F, Shen X (2017). MicroRNA-1291 promotes endometrial fibrosis by regulating the
ArhGAP29-RhoA/ROCK1 signaling pathway in a murine model. Mol Med Rep.

[13] Guo LP, Chen LM, Chen F, Jiang NH, Sui L (2019). Smad signaling coincides with epithelial-mesenchymal transition
in a rat model of intrauterine adhesion. Am J Transl Res.

[14] Yao Y, Chen R, Wang G, Zhang Y, Liu F (2019). Exosomes derived from mesenchymal stem cells reverse EMT via
TGF-β1/Smad pathway and promote repair of damaged
endometrium. Stem Cell Res Ther.

[15] Tulac S, Nayak NR, Kao LC, Waes M Van, Huang J, Lobo S (2003). Identification, characterization, and regulation of the canonical
Wnt signaling pathway in human endometrium. J Clin Endocrinol Metab.

[16] Akhmetshina A, Palumbo K, Dees C, Bergmann C, Venalis P, Zerr P (2012). Activation of canonical Wnt signalling is required for
TGF-β-mediated fibrosis. Nat Commun.

[17] Van Der Horst PH, Wang Y, Van Der Zee M, Burger CW, LJ Blok (2012). Interaction between sex hormones and WNT/β-catenin signal
transduction in endometrial physiology and disease. Mol Cell Endocrinol.

[18] Li D, Li H, Wang Y, Eldomany A, Wu J, Yuan C (2018). Development and characterization of a polarized human endometrial
cell epithelia in an air-liquid interface state. Stem Cell Res Ther.

[19] Ning J, Zhang H, Yang H (2018). MicroRNA-326 inhibits endometrial fibrosis by regulating
TGF-β1/Smad3 pathway in intrauterine adhesions. Mol Med Rep.

[20] Zhang L, Wang M, Zhang Q, Zhao W, Yang B, Shang H (2019). Estrogen therapy before hysteroscopic adhesiolysis improves the
fertility outcome in patients with intrauterine adhesions. Arch Gynecol Obstet.

[21] Yang JH, Chen CD, Chen SU, Yang YS, Chen MJ (2016). The influence of the location and extent of intrauterine
adhesions on recurrence after hysteroscopic adhesiolysis. BJOG.

[22] Chen C, Zhao KN, Masci PP, Lakhani SR, Antonsson A, Simpson PT, Vitetta L (2015). TGFβ isoforms and receptors mRNA expression in breast tumours:
prognostic value and clinical implications. BMC Cancer.

[23] Qian W, Cai X, Qian Q, Zhang W, Tian L (2020). Metastasis-associated protein 1 promotes epithelial-mesenchymal
transition in idiopathic pulmonary fibrosis by up-regulating Snail
expression. J Cell Mol Med.

[24] Yoriki K, Mori T, Kokabu T, Matsushima H, Umemura S, Tarumi Y, Kitawaki J (2019). Estrogen-related receptor alpha induces epithelial-mesenchymal
transition through cancer-stromal interactions in endometrial
cancer. Sci Rep.

[25] Wu RF, Huang ZX, Ran J, Dai SJ, Lin DC, Ng TW (2018). Lipoxin A suppresses estrogen-induced epithelial-mesenchymal
transition via ALXR-dependent manner in endometriosis. Reprod Sci.

[26] Liu Z, Yi L, Du M, Gong G, Zhu Y (2019). Overexpression of TGF-β enhances the migration and invasive
ability of ectopic endometrial cells via ERK/MAPK signaling
pathway. Exp Therap Med.

[27] Cheong Ml, Lai TH, Wu WB (2019). Connective tissue growth factor mediates transforming growth
factor β-induced collagen expression in human endometrial stromal
cells. PLoS One.

[28] Zhang Y, Chen F, Li TC, Duan H, Wu YH (2017). Effects of estradiol at different levels on rabbit endometrial
repair after curettage. J Reprod Med.

[29] Kouzmenko AP, Takeyama K, Ito S, Furutani T, Sawatsubashi S, Maki A (2004). Wnt/beta-catenin and estrogen signaling converge in
vivo. J Biol Chem.

[30] Zhu HY, Ge TX, Pan YB, Zhang SY (2017). Advanced role of hippo signaling in endometrial fibrosis:
implications for intrauterine adhesion. Chin Med J (Engl).

[31] Makena MR, Gatla H, Verlekar D, Sukhavasi S, Pandey MK, Pramanik KC (2019). Wnt/β-Catenin signaling: the culprit in pancreatic carcinogenesis
and therapeutic resistance. Int J Mol Sci.

